# Comparative characteristic of quality of life with patients suffering from juvenile idiopathic arthritis (JIA), attending school and taught at home

**DOI:** 10.1186/1546-0096-9-S1-P106

**Published:** 2011-09-14

**Authors:** TA Shelepina, NY Stepanenko, ES Fedorov

**Affiliations:** 1 Rheumatology Research Institute of the Russian Academy of Medical Sciences, Moscow, Russia

## Objective

To compare the quality of life with patients suffering from JIA, attending school and taught at home.

## Material

Basing on data of 99 patients 14-17 old with JIA boys/girls - 27%/73%, 1- group of children attending school 62 (63%), 2 – 37 patients taught at home; among whom - 20 (group 2A) on medical indications; 17 (group 2B) at will of the patient and parents. 1^st^ group (syst. variant-14%, poly-50%, oligo-20% , SA-16%), 2^nd^ group - (syst*-27%*, poly-49%, oligo-11%., SA-13%), 2-A (*syst-40%*, poly-50%, oligo-5%, CA-5%), 2B- (syst-11%, poly-47%, oligo-18%. SA-24%).

## Methods

Questionnaire: SF-36, EQ-5D, HAQ, CHAQ; Kettel, author's method of social activity, Spielberger-Khanin scale; Visual analogue scale (VAS) – assessment of pain, VAS – global evaluation of health, Well-Being Index (WHO-5)

## Results

VAS of pain ( 23*, 35, 34, 40* mm; p=0,000).

SF-36.**PF** 1^st^ group-77, 2^nd^ group-54, 2A-55, 2B-56; **RP**- 1gr -62, 2 - 48, 2A - 50, 2B - 50); **BP**: 70, 47, 57, 37; p=0.07), **GH**: (57*,43, 52, 37*; *p*=*0.018*; **VT**: *64*, 51, 60,*42 p*=*0.014*; **SF**: 76*, 59, 65, 55*; p=*0.004;***RE**: 77*, 70, 22*, 61; p=0.015; **MH**: 69*, 61, 62, 40*; *p*=*0.035* . * - the difference is statistically reliable.

EQ-5D: 1.5; 1.6; 1.5; 1.3; VAS – global evaluation of health (76 , 57, 52, 64 mm).

HAQ, CHAQ: 0.3* ; 0.9; 1.12*: 0.6.p=0.02

WHO-5 (Norm >13): (15.4; 13.5; 14.2; 13).

Regular communication with equals in age: 1^st^ group -82%; 2^nd^ group - 43%; 2A group - 40%; 2B group 45%

Attendance of cultural events at least once a month in the 1^st^ group - 50%, in the 2^nd^ group, 30%, 2A group - 20%, 2B group -36%.

Communication disorders: 1^st^ group - 68%; 2^nd^ group - 80%, 2A-77%, 2B-72%; fears: 26%, 43%, 46%, 54%. Family stress: 71%, 40%, 38%, 36%.

## Conclusion

The quality of life was higher with children attending school and considerably lower with children groundlessly taught at home.

